# A Cadaveric Study of Variations in Lung Fissures and Drainage Patterns of Pulmonary Veins at the Hilum With Its Clinical Implications

**DOI:** 10.7759/cureus.71909

**Published:** 2024-10-20

**Authors:** C Regina, Kalaivannan J, Santhini Arulselvi Kaliyaperumal

**Affiliations:** 1 Anatomy, Vinayaka Mission’s Medical College, Karaikal, Vinayaka Mission's Research Foundation, Salem, IND

**Keywords:** accessory fissures, atrial fibrillation, lobectomy, lung fissures, pulmonary vein

## Abstract

Introduction

The human lungs are located in the pleural cavity, divided by fissures into lobes, facilitating respiration movements. It acts as a barrier to prevent the spread of infection to adjacent lobes. The pulmonary hilum in each lung contains pulmonary vessels, bronchial vessels, and the bronchus. Pulmonary venous drainage variations are expected to be significant in diagnostic and therapeutic procedures. The present study aims to study the variant pattern of the lung fissures and the drainage pattern of the right and left pulmonary veins at the pulmonary hilum.

Methodology

The descriptive study was conducted on 75 lungs in the Department of Anatomy after obtaining ethical approval from Vinayaka Mission's Medical College and Hospital, Karaikal, India. A few lung specimens were procured from the government hospital, Karaikal. All the lung specimens belonging to adults of unknown age and gender collected over three years were included in our study. Lungs with evident pathological conditions, damage, surgical resections, and metastatic disease were excluded from the study. The pattern of oblique and horizontal fissures and accessory fissures was studied on both the right and left lungs. These fissures were categorized based on the Craig and Walker classification, a widely accepted system in pulmonary anatomy. The hilar structures were dissected on both the right and left lungs, the pulmonary veins were picked up, the different drainage patterns of pulmonary veins at the hilum were studied, and the results were expressed in percentage. In addition, the presence of an accessory vein and anomalous unilateral single pulmonary vein was documented.

Results

Out of 35 right lungs, incomplete and absent oblique fissures were observed in 10 (28.57%) and four (11.43%), and incomplete and absent horizontal fissures were observed in 12 (34.29%) and seven (20%) of the specimens. Out of 40 left lungs, incomplete and absent oblique fissures were observed in 13 (32.5%) and three (7.5%) of the specimens. Superior and inferior accessory fissures were present in five (14.29%) and four (11.4%) of the right lungs, and the left minor fissures were seen in 15 (37.5%) of the specimens.

Different patterns of pulmonary venous drainage were reported in our study, and variations were observed in 52% of the specimens. A single unilateral pulmonary vein was documented in two (5.71%) and 14 (35%), and the accessory vein was noted in seven (20%) and two (5%) of the right and left lungs, respectively. These findings underscore the importance of understanding the variant patterns of pulmonary veins and the presence of accessory veins in the context of cardio-thoracic surgeries.

Conclusions

As revealed by our study, the variations in the pulmonary veins and fissures are paramount in the surgical field. These findings will reassure and instill confidence in surgeons and radiologists, enabling them to diagnose and perform cardio-thoracic surgeries with a strong foundation of anatomical knowledge.

## Introduction

The lungs are the essential respiratory organ in the pleural cavity on either side of the mediastinum. Each lung is half conical in shape and divided into lobes by the presence of fissures. The right lung is divided into three lobes, upper, middle, and lower, by oblique and horizontal fissures, and the left lung is divided by oblique fissures into two lobes, upper and lower. The presence of fissures facilitates the movement of lobes and helps in the uniform expansion of the whole lung during respiration [1]. There are two fissures: major (including oblique and horizontal fissures) and accessory fissures [2]. The major fissure may be "complete," where the lobes are held together at the hilum by bronchi and bronchial vessels, and "incomplete," where the lung parenchyma is fused. The presence of incomplete fissures carries the risk of transmitting infections from one lobe to adjacent lobes through the lung parenchyma [3]. An accessory fissure is a deepened split of varying depths lined by visceral pleura. It is commonly seen in cadaveric specimens and often misinterpreted in imaging techniques. Radiologically, it may appear as a thin white line, which can be frequently mistaken as lung lesions such as basal scars, walls of bulla, and basal pneumothorax [4]. The most common accessory fissures are superior accessory fissures (SAF), inferior accessory fissures (IAF), and left minor fissures (LMF). The incidental finding of accessory fissures during lung resection may cause a potential risk of postoperative surgical complications [2,4].

The mediastinal part of the lung presents a triangular depressed area named the hilum, which allows structures to enter and exit the organ. The pulmonary hilum contains one pulmonary artery, two pulmonary veins (PV) (superior PV (SPV) and inferior PV (IPV)), and a principal bronchus, which varies in number based on their subdivision mode between the two lungs. The hilar structures in both the lungs, from anterior to posterior, are PV, pulmonary arteries, and bronchus [1]. PV anatomy and drainage patterns are essential for diagnostic and therapeutic procedures. There are four PV with two superior and two inferior drains into the left atrium on either side. The right SPV drains the right upper and middle lobe, and the right IPV drains the right lower lobe. Likewise, the left SPV drains the left upper lobe and lingula, and the left IPV drains the left lower lobe of the lung [1]. This regular drainage pattern is commonly seen in 82% of the population [5].

Much literature is available about the variations in pulmonary venous drainage radiologically and as a case report in cadaveric specimens, and it has been reported that the variations were seen in 36% of the cases [5,6]. Recent studies have mentioned that PV play a crucial role in the pathophysiology of paroxysmal atrial fibrillation (AF) due to myocardial sleeves, which trigger ectopic depolarization. Therapeutic procedures like radiofrequency ablation have been performed recently to treat AF [7]. Many authors have documented the variations in lung morphology on bronchi, pulmonary arteries, and fissures worldwide. Still, a gap exists in detailed studies about the drainage pattern of PV at the hilum in cadaveric specimens. Since the PV and lung fissures are crucial in diagnostic and therapeutic approaches, mapping such structures is essential, enabling radiologists and cardio-thoracic surgeons to diagnose and manage cardio-pulmonary cases clinically [8].

Aims and objectives of the study

The aims and objectives of the study are to determine the variations in the drainage pattern of right and left PV at the pulmonary hilum, to classify the presence of fissures in right and left lungs according to the Craig and Walker classification, and to detect the presence of accessory fissures in both right and left lungs with its clinical implications. These findings significantly impact the diagnosis and management of cardio-pulmonary cases and enhance the safety and efficacy of therapeutic procedures.

## Materials and methods

The descriptive study was conducted in the Department of Anatomy, Vinayaka Mission's Medical College and Hospital (VMMC & H), Karaikal, India. Ethical approval was obtained from the Institutional Ethical Committee of VMMC & H, Karaikal, India (approval no: IEC/VMMCH/2024/APR/90 dated April 16, 2024). In collaboration with the Department of Anatomy, VMMC & H, a few lung specimens were procured from the government hospital, Karaikal. All the lung specimens belonging to adults of unknown age and gender collected over three years were included in our study. Lungs with evident pathological conditions, damage, surgical resections, and metastatic disease were excluded from the study. The study samples included 75 lung specimens that met the aforementioned inclusion and exclusion criteria, out of which 35 were right and the remaining 40 lungs were left. The pattern of absent and incomplete oblique and horizontal fissures and accessory fissures, such as SAF, IAF, and LMF, was meticulously studied on both the right and left lungs. Based on the Craig and Walker classification, oblique and horizontal fissures have been categorized, and the results are documented in percentages. The hilar structures were dissected, and the PV with their tributaries were exposed with meticulous care using blunt forceps on both the right and left lungs. The drainage pattern of the SPV and IPV tributaries was studied, and the results are expressed in percentages. In addition, an accessory vein and anomalous unilateral single PV (AUSPV) were documented.

## Results

**Figure 1:**
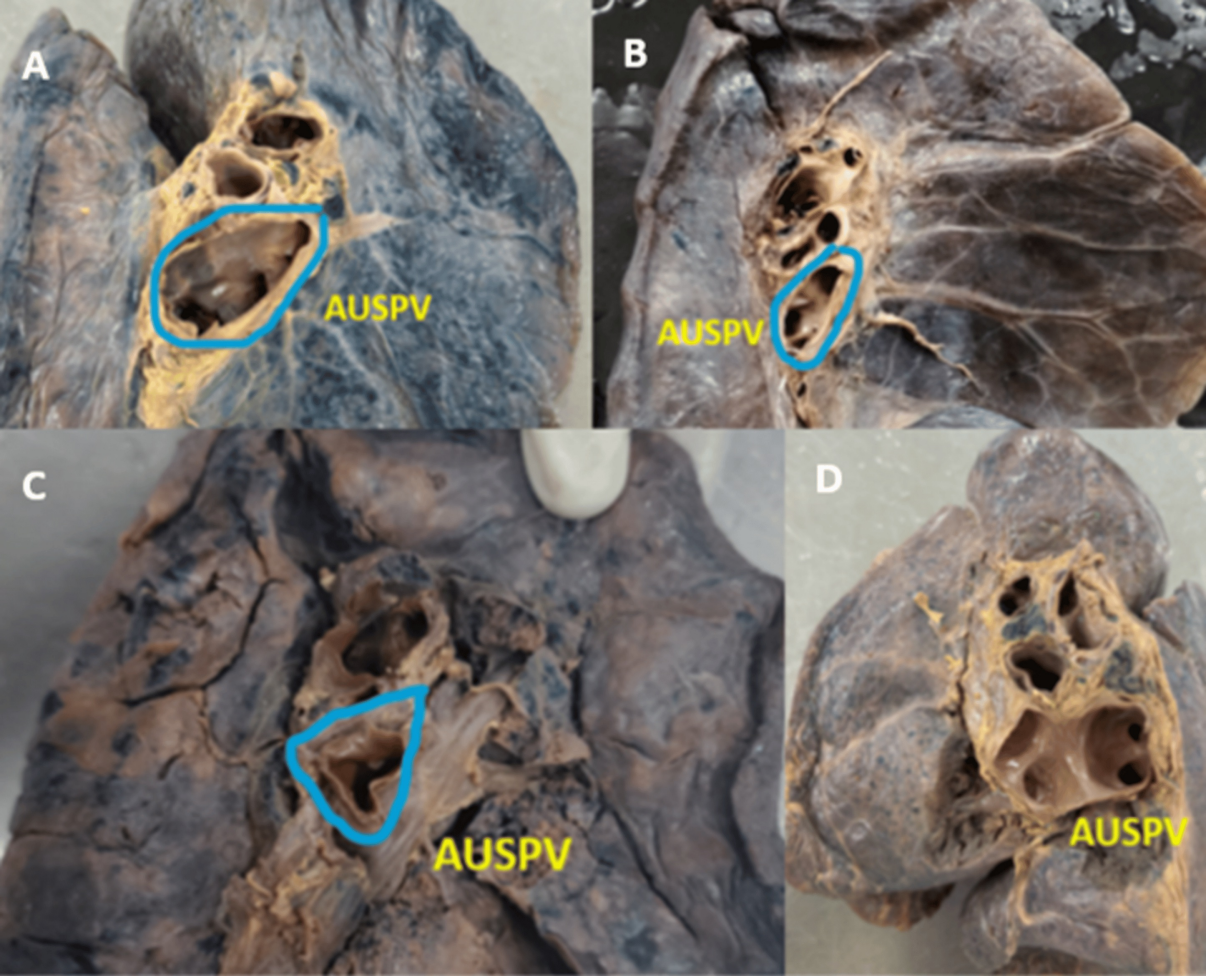
(A-D) Cadaveric image showing the presence of an anomalous unilateral single pulmonary vein at the hilum of the left and right lungs AUSPV: anomalous unilateral single pulmonary vein

**Figure 2:**
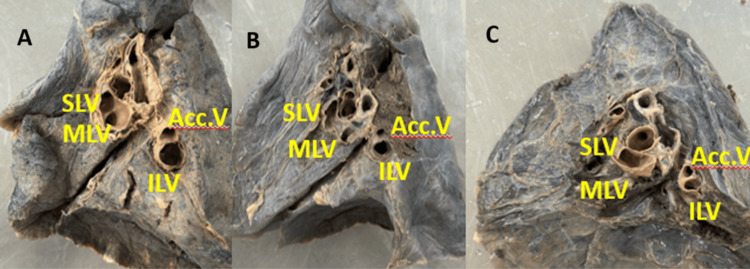
(A-C) Cadaveric images showing the presence of an accessory vein in the hilum of the right lung SLV: superior lobar vein; MLV: middle lobar vein; ILV: inferior lobar vein; Acc. V: accessory vein

**Figure 3:**
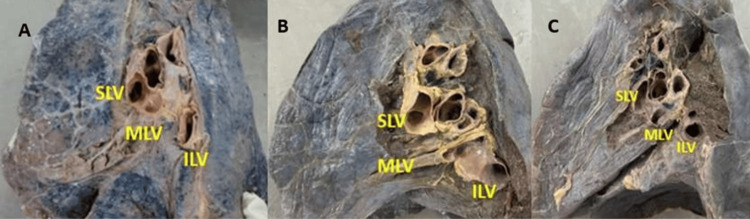
Cadaveric image showing the pulmonary venous drainage pattern at the hilum of the right lung. (A) SLV joins with MLV drained as SPV; (B) MLV joins with ILV drained as IPV; (C) SLV, MLV, and ILV drained separately at the hilum of the right lung SLV: superior lobar vein; MLV: middle lobar vein; ILV: inferior lobar vein; SPV: superior pulmonary vein; IPV: inferior pulmonary vein

**Figure 4:**
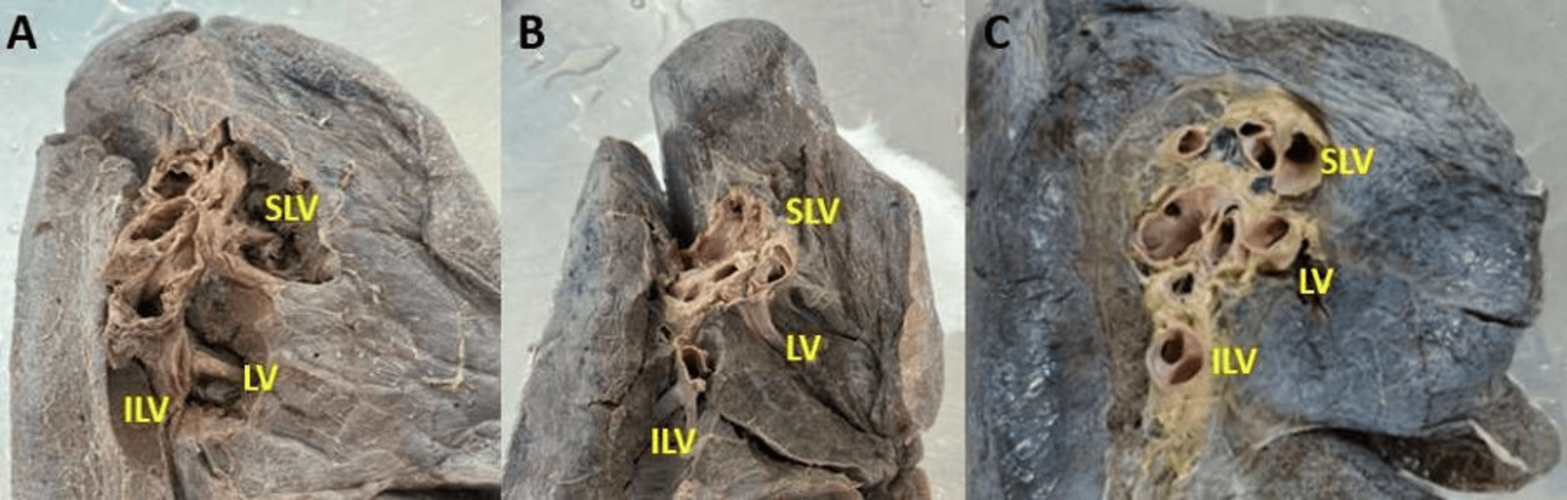
Cadaveric image showing the pulmonary venous drainage pattern at the hilum of the left lung. (A) LV joins with ILV drained as IPV; (B) SLV joins with LV drained as SPV; (C) SLV, LV, and ILV drained separately at the hilum of the left lung LV: lingular vein; ILV: inferior lobar vein; IPV: inferior pulmonary vein; SLV: superior lobar vein; SPV: superior pulmonary vein

**Figure 5:**
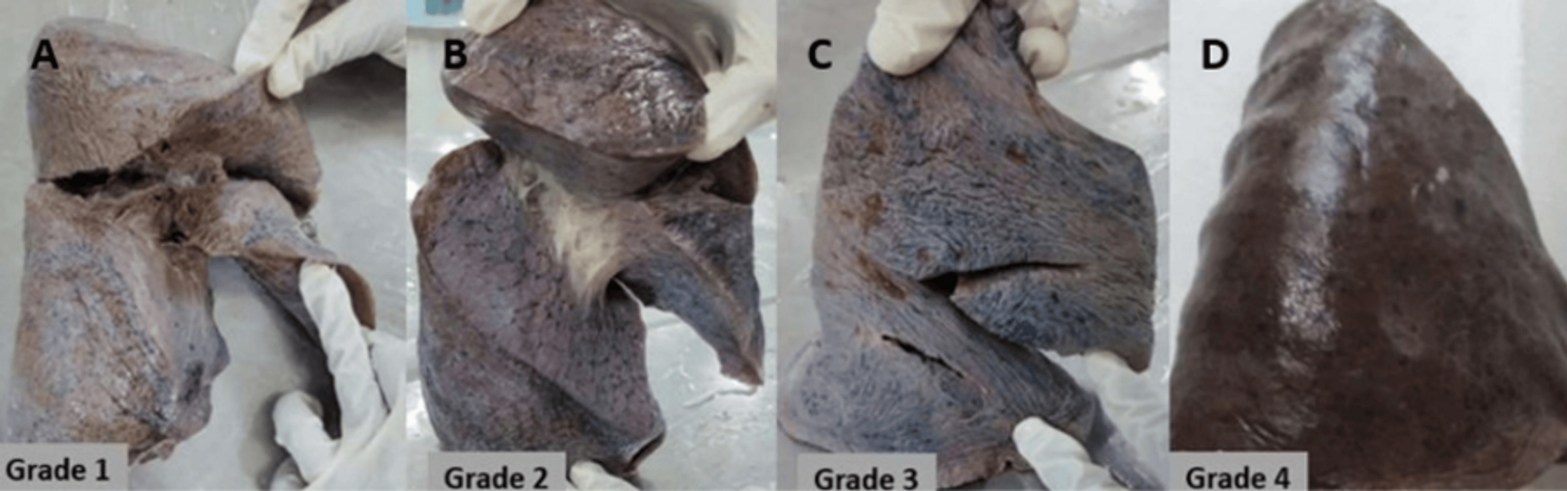
(A-D) Cadaveric lung showing the different grades of horizontal and oblique fissures of the right lung based on the Craig and Walker classification

**Figure 6:**
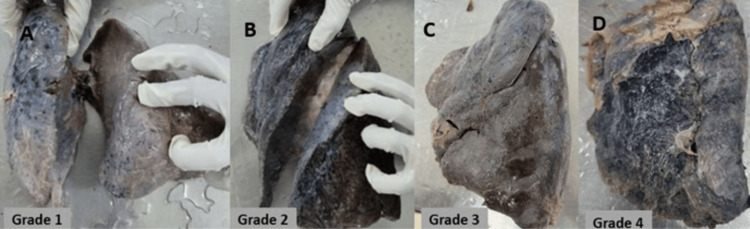
(A-D) Cadaveric lung showing the different grades of oblique fissure of the left lung based on the Craig and Walker classification

**Figure 7:**
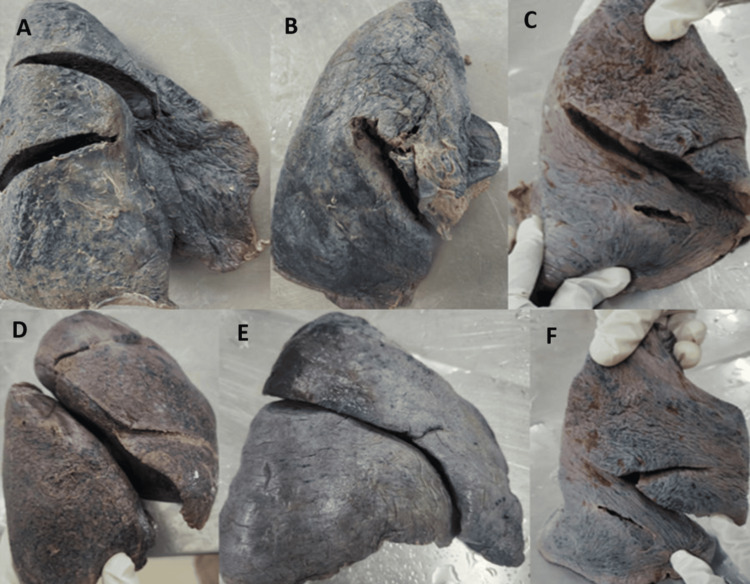
All the right lungs (A-C) show incomplete oblique fissure, and the below three lung images (D-F) show incomplete horizontal fissure

**Figure 8:**
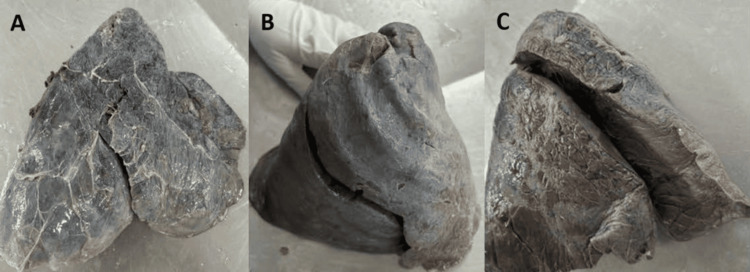
(A-C) Right lung with absent horizontal fissure presents with two lobes

**Figure 9:**
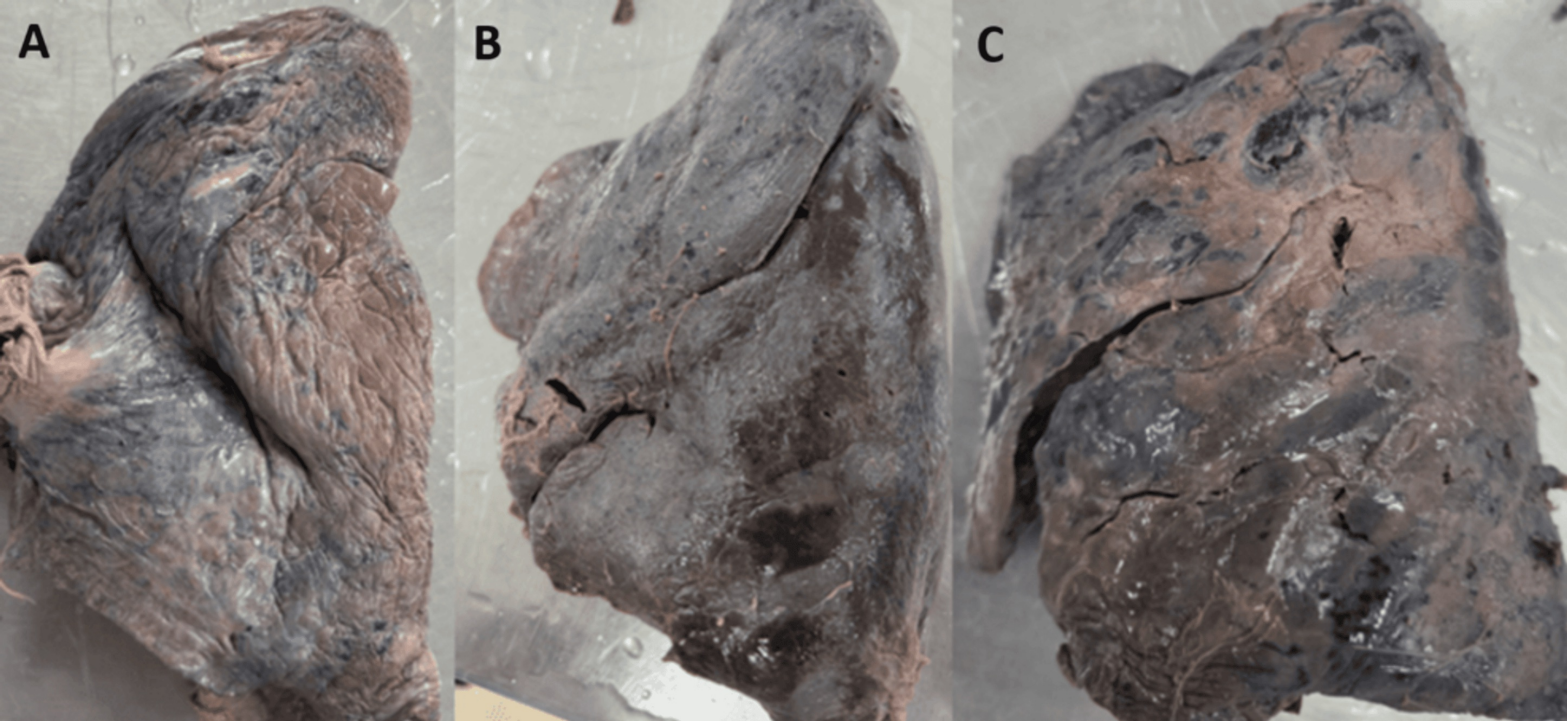
(A-C) Left lung with incomplete oblique fissure

**Figure 10:**
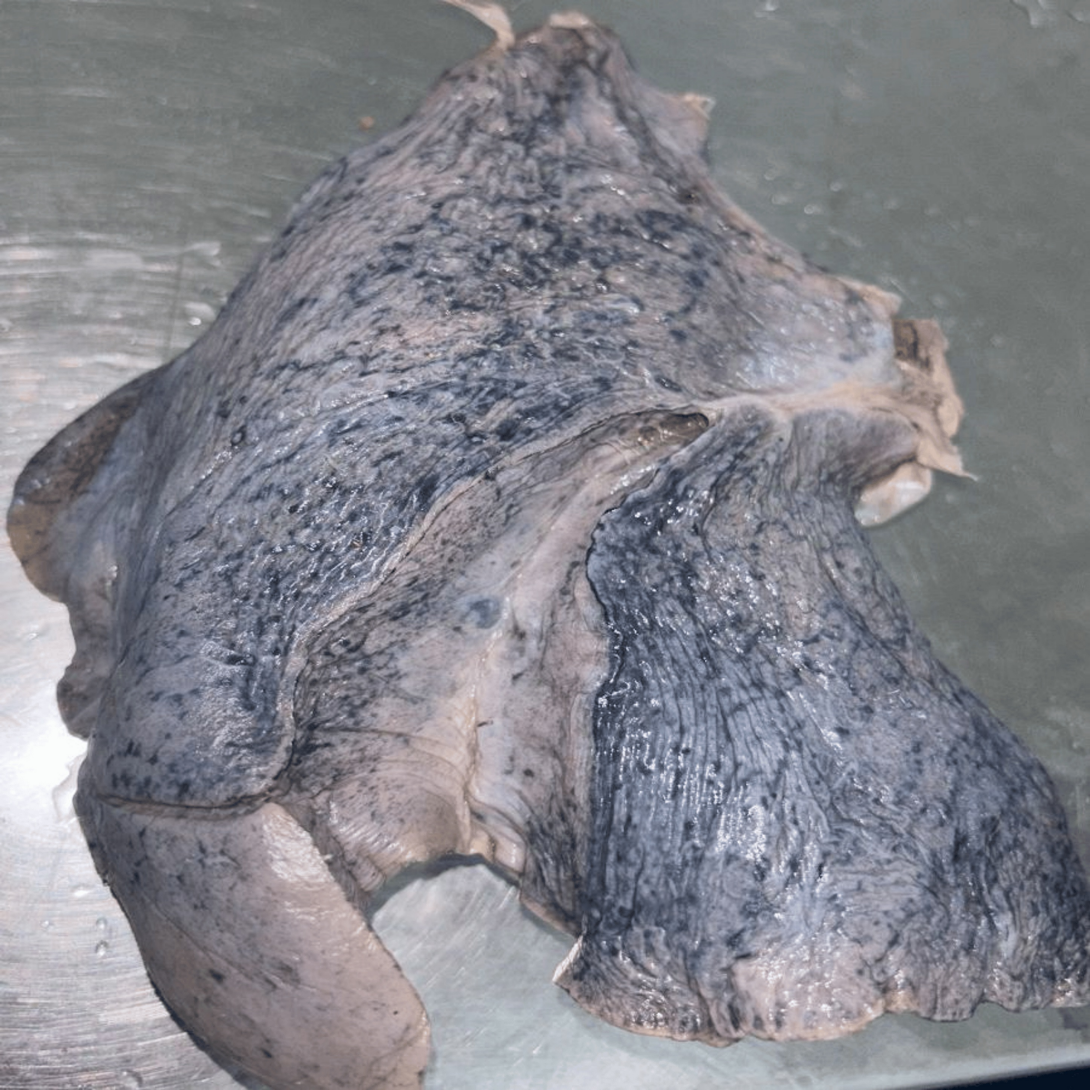
Left lung with accessory fissure presents with three lobes

**Figure 11:**
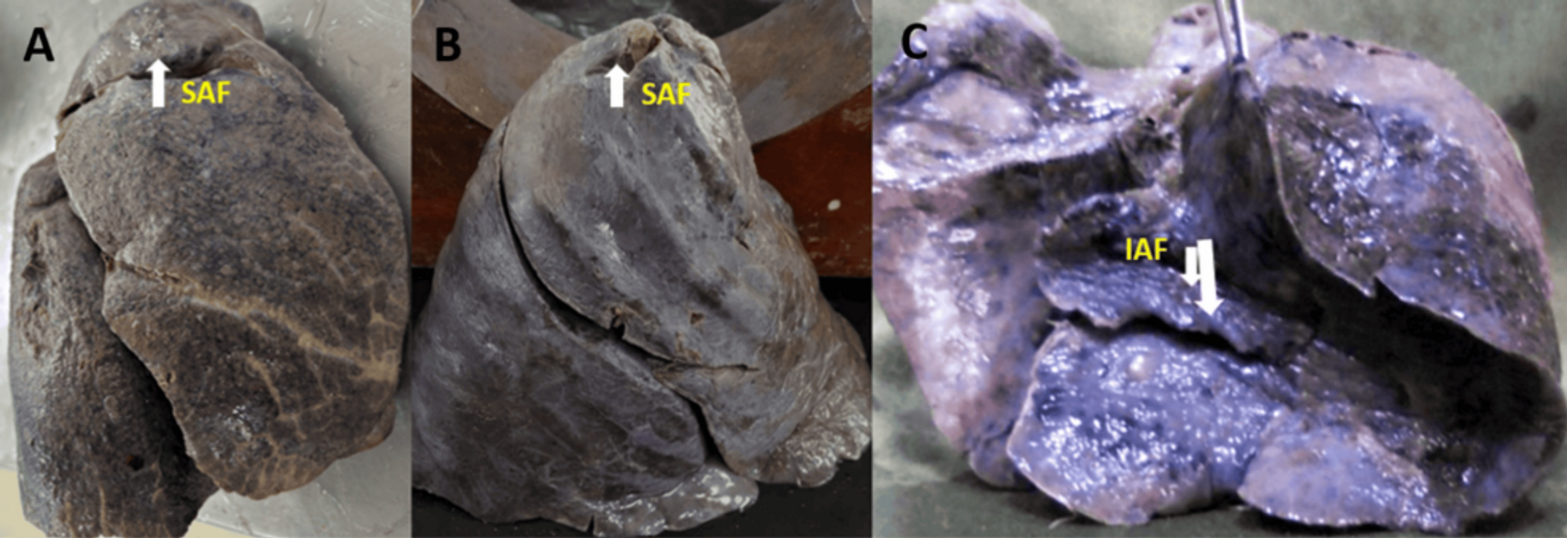
(A-C) Right lung with the presence of an accessory fissure SAF: superior accessory fissure; IAF: inferior accessory fissure

**Figure 12:**
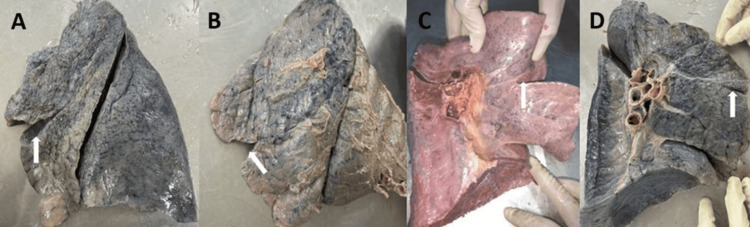
(A-D) Left lung showing the presence of left minor fissure (white arrow)

Of the 75 lung specimens, 35 were right and 40 were left lungs. The study's findings on the drainage pattern of the PV and the pattern of major and accessory fissures on both right and left lungs, including the Craig and Walker classification, are documented in Tables 1-4 and Figures 1-12. These findings provide a comprehensive understanding of the variations in lung morphology and PV drainage patterns, which is crucial for diagnostic and therapeutic procedures in cardio-pulmonary cases.

**Table 1:** Drainage pattern of right pulmonary veins at the hilum of the right lung SLV: superior lobar vein; MLV: middle lobar vein; ILV: inferior lobar vein; SPV: superior pulmonary vein; IPV: inferior pulmonary vein; AUSPV: anomalous unilateral single pulmonary vein

Drainage pattern at right hilum	Number (n-35)/frequencies (%)
The SLV and the MLV drained as the SPV, and the IPV drained independently	16 (45.72)
MLV and ILV drained as IPV, and SLV drained independently	8 (22.86)
SLV drained as SPV, MLV drained independently, and ILV drained as IPV	2 (5.71)
Presence of accessory vein	7 (20)
AUSPV	2 (5.71)

**Table 2:** Drainage pattern of left pulmonary veins at the hilum of the left lung SLV: superior lobar vein; LV: lingular vein; ILV: inferior lobar vein; SPV: superior pulmonary vein; IPV: inferior pulmonary vein; AUSPV: anomalous unilateral single pulmonary vein

Drainage pattern at left hilum	Number (n-40)/frequencies (%)
SLV and LV drained as SPV, and IPV drained independently	17 (42.5)
SLV drained independently, and LV and ILV drained as IPV	5 (12.5)
SLV drained as SPV, LV drained independently, and ILV drained as IPV	2 (5)
Presence of accessory vein	2 (5)
AUSPV	14 (35)

**Table 3:** Incidence of oblique and horizontal fissures in the right and left lungs based on the Craig and Walker classification

Lung	Fissure	Grade 1	Grade 2	Grade 3	Grade 4
		Number (%)	Number (%)	Number (%)	Number (%)
Right lung (35)	Oblique fissure	10 (28.57)	11 (31.43)	10 (28.57)	4 (11.43)
	Horizontal fissure	11 (31.43)	5 (14.28)	12 (34.29)	7 (20)
Left lung (40)	Oblique fissure	14 (35)	10 (25)	13 (32.5)	3 (7.5)

**Table 4:** Incidence of major and accessory fissure variations in the right and left lungs

Lung	Abnormalities in the fissures		Number (n)/frequency (%)
Right lung (35)	Oblique fissure	Absent	4 (11.43)
		Incomplete	10 (28.57)
	Horizontal fissure	Absent	7 (20)
		Incomplete	12 (34.29)
	Accessory fissure	Superior accessory fissure	5 (14.29)
		Inferior accessory fissure	4 (11.43)
Left lung (40)	Oblique fissure	Absent	3 (7.5)
		Incomplete	13 (32.5)
	Accessory fissure	Superior accessory fissure	-
		Inferior accessory fissure	-
		Left minor fissure	15 (37.5)

Variant drainage patterns of the right PV are depicted in Figures 1D, 2 and Figures 3A-3C, and the results are tabulated in Table 1. The most common drainage pattern was where the superior lobar vein (SLV) joins with the middle lobar vein (MLV), drained as the SPV (45.72%). In 22.86% of the specimens, the MLV joins with the inferior lobar vein (ILV), drained as the IPV at the hilum of the right lung. In 5.7% of the specimens, all three veins (SLV, MLV, and ILV) drained independently at the hilum of the right lung. The AUSPV was seen in 5.7% of the specimens, and the accessory vein was in 20%. The variant drainage pattern of the left PV is depicted in Figures 1A-1C and Figures 4A-4C, and the results are tabulated in Table 2. The most common drainage pattern was where the SLV joins with the lingular vein (LV) drained as SPV (42.5%), and in 12.5% of the specimens, the LV joins with the ILV drained as IPV at the hilum of the left lung. In 5% of the specimens, all three veins (SLV, LV, and ILV) drained independently at the hilum of the left lung. The presence of AUSPV was seen in 35%, and the accessory vein was observed in 5% of the specimens.

Craig and Walker, in 1997, proposed an anatomical classification to determine the presence and completeness of the fissures on both the right and left lungs [9]. Based on the degree of fissures, grade 1 denotes a complete fissure with separate lobes, grade 2 denotes a complete visceral cleft with parenchymal fusion at the base of the fissure, grade 3 denotes visceral cleft evident for a part of the fissure, and grade 4 denotes a complete fusion of lobes with no evidence of fissure. The different grading of pulmonary fissures based on the Craig and Walker classification is depicted in Figures 5A-5D and Figures 6A-6D. Grading was done for both oblique and horizontal fissures in the right lung; the results are documented in Table 3. Grade 1 was observed in 28.57% and 31.43%, grade 2 in 31.43% and 14.28%, grade 3 in 28.57% and 34.29%, and grade 4 in 11.43% and 20% of the specimens. In the left lung, grade 1 was seen in 35%, grade 2 in 25%, grade 3 in 32.5%, and grade 4 in 7.5% of the specimens.

The major fissures of the right and left lung may be absent or incomplete, as depicted in Figures 7-10, and the results are tabulated in Table 4. In the right lung, absent and incomplete oblique fissures were seen in 11.4% and 28.57% of the specimens, and absent and incomplete horizontal fissures were seen in 20% and 34.29%. Similarly, absent and incomplete oblique fissures were observed in 7.5% and 32.5% of the specimens in the left lung. The accessory fissures were observed in both the right and left lungs, as shown in Figures 11, 12, and the results are documented in Table 4. The SAF and IAF were absent in the left lung and present in the right lung, with 14.29% and 11.4%. In the left lung, a minor fissure was seen in 37.5% of the specimens. Pre-operative knowledge about the variant pattern of major and accessory fissures helps localize pulmonary parenchymal pathologies and prevents postoperative complications like bleeding and air leaks [3].

## Discussion

PV play an essential role in lung segmental anatomy as it determines the surgical borders of bronchopulmonary segments. Knowledge about its anatomical variations is necessary for performing thoracic procedures like pulmonary resections and lobectomies. In recent years, the introduction of minimally invasive techniques like video-assisted thoracoscopic surgery (VATS) has still been challenging for surgeons in the operating field as they need a better understanding of thoracic anatomy. In the case of cardio-thoracic surgeries, incidental damage of the pulmonary vessels due to their variations may result in catastrophic bleeding [10]. In our present study, we have observed two variant anomalies: an AUSPV and a more significant number of PV, including accessory veins on the right and left lungs. The AUSPV is also called a conjoined or common vein, where SPV and IPV unite on the same side and present as a single unilateral PV at the pulmonary hilum. It is more commonly seen on the left side than on the right side [11]. In our study, we reported 5.71% and 35% of AUSPV in the hilum of the right and left lungs. Healey Jr., in 1952, reported the presence of AUSPV in 53 left lungs and seven right lungs out of 251 lung specimens, with an incidence of 23.9% [12]. Many investigators have observed AUSPV in both lungs; the results are compared in Table 5. Shukla et al. observed 3.4% and 17.2% of AUSPV in the right and left lungs, and Polaczek et al. in 11.11% of the left lungs [13,14]. In 12% of the right lungs, AUSPV was observed by Sultana and Chandrupatla [15]. Compared with other studies, we have reported more AUSPV, with 35% in the left lungs.

**Table 5:** Comparative incidence of an anomalous unilateral single pulmonary vein (AUSPV) among various studies CT: computerized tomography; RL: right lung; LL: left lung

Studies	No. of specimens	Right lung	Left lung
		Number/frequency (%)	Number/frequency (%)
Marom et al., 2003 [6]	201 CT chest	3 (2)	21 (10)
Saha and Srimani, 2018 [10]	RL: 49; LL: 54	3 (6.12)	5 (9.26)
Healey Jr., 1952 [12]	251 specimens	7 (2.78)	53 (21.11)
Shukla et al., 2012 [13]	-	1 (3.4)	5 (17.2)
Polaczek et al., 2020 [14]	135 CT chest	1 (<1)	15 (11.11)
Sultana and Chandrupatla, 2017 [15]	40 cadaveric lungs	5 (12)	-
Present study, 2024	RL: 35; LL: 40	2 (5.71)	14 (35)

Patients with AUSPV are asymptomatic and are incidentally found during imaging studies and dissections. It does not require surgery because no vascular shunt is produced due to normal drainage into the left atrium. The AUSPV may be strongly associated with pulmonary and vascular malformations. AUSPV has standard features with scimitar syndrome, which shares a similar pathogenesis. However, it has to be distinguished from scimitar syndrome, in which the vein drains into the inferior vena cava after piercing the diaphragm, and it is frequently associated with atrial septal defect, hypogenetic right lung syndrome, and anomalous systemic arterial supply to the lung [16]. Sometimes, it may be confused with other entities like pulmonary arteriovenous malformations, pulmonary varix, and nodules [16-18]. Differentiating AUSPV from other pathologies is vital, as the anomalous veins do not require treatment. The accessory vein, the supernumerary vein, is separate from SPV or IPV, with independent drainage into the left atrium. It is more commonly seen on the right side, and its prevalence was reported in 30% of cases [6]. These veins are usually small and often drain only one pulmonary segment, particularly the superior segment of the lower lobe [5]. Very few authors have reported the presence of accessory veins [5,10]. Shukla et al. reported 6.9% and 3.5% of accessory veins in both right and left lung specimens [13]. Our study observed more accessory veins in the right lung (20%) than in the left (5%). If more than one number of PV drains anomalously into the left atrium, the volume is sufficient to produce right ventricular diastolic overload [10]. In addition, a variable number of PV could remarkably affect the success rate of radiofrequency ablation in the case of AF, as the ectopic foci may go untreated, failing the procedures with the recurrence rate [6].

The different pattern of pulmonary venous drainage was observed in our study, and the results are documented in Tables 1, 2. The standard variant observed in pulmonary venous drainage is the MLV emptying into the IPV, which carries surgical importance. Many kinds of literature have mentioned the MLV variations, and the results are tabulated in Table 6. Sugimoto et al. and Subotich et al. have observed MLV emptying into IPV while performing surgery for various lung pathologies [19,20]. Rajeshwari and Ranganath and Yazar et al. have reported 11.53% and 3.3% of MLV emptying into IPV on the right lung [21,22]. In patients with an anatomical variation of the MLV emptying into IPV, accidental damage to the IPV during the lobectomy procedure causes blockage of venous return from the MLV due to its short trunk [14]. It may also lead to life-threatening postoperative complications like lung edema, infections, and respiratory distress [19]. Similarly, LV emptying into IPV in the left lung is a rare variant that few investigators have reported, with a frequency of 2.5% [23]. We have observed similar variations in five specimens with an incidence of 12.5%.

**Table 6:** Prevalence of variant drainage pattern of the middle lobar vein (MLV) in both right and left lungs among various studies IPV: inferior pulmonary vein; LV: lingular vein; CT: computerized tomography; ILV: inferior lobar vein

Studies	Results
Cronin et al., 2007 [5]	Right MLV emptying into IPV in 5.5% of 200 CT chest cases
Polaczek et al., 2020 [14]	Right MLV emptying into IPV in 2.96% out of 135 CT chest cases
Sugimoto et al., 1998 [19]	Observed 2 cases of right MLV emptying into IPV during surgery for lung carcinoma
Subotich et al., 2009 [20]	Observed 4 cases (17.4%) of MLV emptying into IPV during lung resection of various pathologies
Rajeshwari and Ranganath, 2012 [21]	Of 26 lung specimens, 11.53% of MLV emptied into IPV on the right lung
Yazar et al., 2002 [22]	A similar variation was observed on the right lung, with 3.3% of MLV joining with ILV out of 30 specimens
Yamashita et al., 2000 [23]	On the right lung, MLV is emptying into IPV at 4.8%. On the left lung, LV emptying into IPV with 2.5%
Present study, 2024	On the right lung, MLV emptying into IPV at 23%. On the left lung, LV emptying into IPV at 12.5%

The embryological cause is one of the main reasons for pulmonary venous anomalies. During the fourth week of development, a common PV arises from the posterosuperior wall of the primitive left atrium. The initial route of pulmonary venous drainage was established by splanchnic venous plexus after communicating with systemic cardinal and umbilical-vitelline veins. Later, single PV grows toward the bronchial buds, anastomosing with the splanchnic venous plexus surrounding the lung tissue. PV separates from the splanchnic plexus and gets incorporated within the left atrium, where it branches out to give four separate openings of PV [24]. Failure to separate PV from splanchnic plexus and communication between the PV and cardinal or umbilical-vitelline veins remain patent, which may result in anomalies like anomalous pulmonary venous drainage (APVD) [13]. Atypical incorporation of common PV into the left atrium results in abnormal PV [25]. Failure of PV branching during absorption into LA may form a single unilateral PV [12].

In recent years, much literature has reported that pulmonary venous morphology plays a vital role in the pathogenesis of AF due to the extension of myocardial sleeves from the left atrium to the proximal part of PV [7]. The length of sleeves in PV may vary; SPV tends to be more arrhythmogenic because it has a longer muscular sleeve than IPV, and it is thickest at the veno-atrial junction of the left SPV [5]. The frequency of AF was found to be more common in patients with independent drainage of MLV and accessory veins into the LA [21,22]. Invasive procedures like radiofrequency ablation have been done to correct AF by eliminating the electrical stimulators from the PV [6]. A patient with AF carries a high risk of developing 20% to 25% of strokes [7]. Pulmonary venous drainage variations could often affect therapeutic procedures' success rate. So, it is essential to do pre-ablation imaging studies to detect any variant vein and to determine the pattern of pulmonary venous drainage. Yamashita et al., in 2000, mentioned that the PV would not interfere with disease recurrence or survival. However, it carries a high risk of spreading circulating tumor cells, which must first be interrupted during lobectomy to prevent metastasis. Due to variable pulmonary vasculature arrangement at the hilum, sequential interruption of the vessels might be a hurdle for surgeons in an operating field [23]. It is important to note that PV is often associated with potential misinterpretation of radiographic images due to anatomical variations. The dimensions of the hilar structures were primarily described by the pulmonary arteries with little contribution by the SPV. Therefore, anatomists and radiologists must know about the variant pulmonary vessels, which would be helpful for academic teaching and diagnostic and therapeutic procedures [10].

Embryological basis of accessory fissure formation

During the fifth week of the embryonic period, the laryngotracheal diverticulum arises from the ventral part of the foregut, forming the respiratory tree and the parenchyma. The outpouching gives two lung buds, the right and left, which undergo further division to give lobar and segmental bronchi. By the seventh week, bronchopulmonary segments form and invade the surrounding splanchnopleuric mesoderm [24]. In the initial period, bronchopulmonary segments are separated by spaces; later, they get obliterated except along two planes, giving rise to significant fissures: oblique and horizontal fissures [2]. Defective obliteration of major fissures results in either complete or incomplete fissures, and non-obliteration of spaces between the bronchopulmonary segments is known as accessory fissures [3,26].

Based on the Craig and Walker classification, several authors have reported anomalous fissures and lobes in both lungs. Other investigators observed similar types of grading of fissures, and the results are compared in Table 7. The prevalence of absence of the horizontal fissures was comparatively higher than the absent oblique fissures in both the right and left lungs in all previous studies, including the present study [3,27]. Absent oblique fissures of the left lung were reported in all previous studies, including the present study [3,8,27]. As observed from the different studies, a complete fissure with separate lobes and an absent and incomplete horizontal fissure are common variations. Variations in the pulmonary major and accessory fissure were documented by many investigators in different populations, and the results are compared in Tables 8, 9. Medlar did a study on the American population and found a more significant number of absent horizontal fissures, with 45.2% in the right lungs [28]. In Nepalese populations, more incomplete horizontal and oblique fissures were observed in both the right and left lungs, which coincides with our present study results [29]. We have observed seven cases of absent horizontal fissures with two lobes on the right lung with an incidence of 20%, and on the left lung, a single lung presents with three lobes due to accessory fissures. Recognition of accessory fissures indicated the persistence of embryonic fissures. It acts as a natural barrier against the spread of infections. The most common accessory fissures are the SAF, which demarcates the superior segment; the IAF, which demarcates the medial basal segment; and the LMF, which separates the lingula [4]. Many authors have reported that IAF is more commonly seen than SAF in the right lungs, which coincides with our study results [26,27,29]. Very few studies have observed SAF and IAF in the left lung, which are absent in our present study [26]. Many investigators reported the LMF, and the results are compared in Table 9. We have documented more LMF, with 37.5%, than other studies.

**Table 7:** Comparative incidence of lung fissures among various studies based on the Craig and Walker classification RL: right lung; LL: left lung; OF: oblique fissure; HF: horizontal fissure

Grades	Fissure in specimens	Craig and Walker, 1997 [9]	Manicka Vasuki et al., 2019 [3]	Thapa and Desai, 2016 [8]	Magadum et al., 2015 [27]	Present study, 2024 (RL: 35; LL: 40)
1	RL: OF	53.3%	42.5%	70%	30%	28.57%
	RL: HF	20%	17.5%	30%	35%	31.42%
	LL: OF	13.3%	32.5%	13%	50%	35%
2	RL: OF	-	7.5%	10%	32.5%	31.43%
	RL: HF	-	30%	20%	32.5%	14.29%
	LL: OF	-	15%	10%	30%	25%
3	RL: OF	36.6%	42.5%	20%	27.5%	28.57%
	RL: HF	63.3%	27.5%	30%	20%	34.29%
	LL: OF	46.6%	32.5%	10%	12.5%	32.5%
4	RL: OF	0	17.5%	-	10%	11.43%
	RL: HF	16%	25%	20%	12.5%	20%
	LL: OF	0	17.5%	15%	7.5%	7.5%

**Table 8:** Comparative incidence of anatomical variations of the lung fissures in different populations RL: right lung; LL: left lung

Authors	No. of lungs	Right lung				Left lung	
		Oblique fissure		Horizontal fissure		Oblique fissure	
		Absent	Incomplete	Absent	Incomplete	Absent	Incomplete
Mutua et al., 2021, Kenya [2]	RL: 38; LL: 32	0	36.8%	10.5%	42.1%	0	34.4%
Wahengbam et al., 2019, India [26]	RL: 42; LL: 37	7.14%	42.86%	19.05%	61.90%	2.70%	40.54%
Medlar, 1947, USA [28]	-	4.8%	25.6%	45.2%	17.1%	7.3%	10.6%
Sudikshya et al., 2018, Nepal [29]	RL: 23; LL: 27	0	30.43%	10.53%	42.11%	0	34.38%
Present study, 2024	RL: 35; LL: 40	11.43%	28.57%	20%	34.29%	7.5%	32.5%

**Table 9:** Incidence of accessory fissure among various studies SAF: superior accessory fissure; IAF: inferior accessory fissure; LMF: left minor fissure; RL: right lung; LL: left lung

Authors	No. of specimens	Right lung		Left lung		
		SAF	IAF	SAF	IAF	LMF
Manicka Vasuki et al., 2019 [3]	RL: 40; LL: 40	7.5%	5%	10%	12.5%	2.5%
Wahengbam et al., 2019 [26]	RL: 42; LL: 37	7.14%	21.43%	2.70%	43.24%	29.73%
Magadum et al., 2015 [27]	RL: 40; LL: 40	2.5%	5%	7.5%	-	-
Sudikshya et al., 2018 [29]	RL: 23; LL: 27	4.4%	5%	-	3.7%	29.6%
Present study, 2024	RL: 35; LL: 40	14.29%	11.43%	-	-	37.5%

Gradation of fissures is surgically necessary for ligating the pulmonary vessels and segments during lobectomy procedures [27]. The surgeon's approach through the depth of grade 1 oblique fissure is to ligate the vessels and bronchi during surgeries to prevent postoperative complications like bleeding. On the other hand, a patient with a grade 1 oblique fissure has the chance of undergoing torsion while performing a lobectomy [8]. Therefore, the pre-operative identification of variant pulmonary fissures is not just a step but a crucial one, essential before lobectomies to prevent complications like bleeding and air leaks, which may require further procedures like stapling or pericardial sleeves [3]. Accessory fissure helps in the precise localization of pulmonary parenchymal lesions and differentiate pleural pathology from parenchymal disease. Thus, it helps in the accurate localization of lesions and the characterization of diseases [2]. It may sometimes be misdiagnosed radiologically as linear atelectasis, pleural scars, or bulla due to its thick sections and orientation in imaging studies [4,26]. Incomplete fissures may alter the course of a few clinical conditions like pneumonia, pleural effusion, and lung carcinoma. These conditions are restricted to one particular lobe, which tends to spread to the adjacent lobes through parenchyma due to its incompleteness [30].

Limitations of the study

The current study encompassed a limited number of lung specimens, owing to the nature of variations, which could limit the generalizability of our observations to a more significant population. The authors propose a study in the future involving a more substantial number of specimens, leading to a better understanding and representation of the variations. Even though the author has highlighted the variations in pulmonary venous drainage and the fissures, we fail to study the abnormalities of bronchial and pulmonary arteries associated with incomplete and accessory fissures, which are critical in lung surgeries.

## Conclusions

The present study concludes with anatomical variations in pulmonary venous drainage and pulmonary fissures in cadaveric specimens. The data obtained in this study would be additional information to the existing document in our populations. In this study, we have observed pulmonary venous variations with an incidence of 52% in the cadaveric lungs. Our study reports many absent and incomplete horizontal fissures compared to other fissures. Compared with previous studies, the current study showed a wide range of differences in the fissures among different populations. Variations in the pulmonary fissures help better understand the spread of disease within the lung parenchyma. Prior knowledge about the variations in pulmonary venous drainage and fissures is essential for academic purposes, clinical diagnosis, interpretation of radiological images, and planning of surgical procedures like radiofrequency ablation and pulmonary resections to reduce mortality and morbidity.
